# Energy potential, health benefits, antinutrient reduction methods, and nutritional properties of Indian millets: a review

**DOI:** 10.3389/fnut.2026.1676670

**Published:** 2026-02-11

**Authors:** N. R. Gatkal, M. S. Shelke, S. M. Nalawade, M. S. Deshmukh, Ramesh K. Sahni, Kateřina Beňová, Julie Liška

**Affiliations:** 1 Department of Farm Machinery and Power Engineering, Dr. Annasaheb Shinde College of Agricultural Engineering and Technology, MPKV, Rahuri, Ahmednagar, Maharashtra, India; 2 Department of Soil Science and Agricultural Chemistry, College of Agriculture, Vasantrao Naik Marathwada Krushi Vidyapeeth, Parbhani, India; 3 Central Institute of Agricultural Engineering, Bhopal, Madya Pradesh, India; 4 VSB-Technical University of Ostrava, CEET, ENET Centre, Ostrava-Poruba, Czechia

**Keywords:** dietary diversification, food and nutritional security, functional food, millet, nutritional characteristics, processing and value addition

## Abstract

Millet production has significantly increased to fulfill the nutritional needs of the increased population across the globe. Around the world, millions of people suffer from shortages of food and hunger. In the last few years, food supply has been influenced by many factors, such as changes in climate, increased population, and a slowing economy. Furthermore, many countries face undernutrition and overnutrition problems. Achieving nutritional and food security requires a transformative shift in the agricultural sector. Providing everyone with access to cheap, healthy, and affordable food as well as a nutritious diet is one way to reach our goal. The present study uses preferred reporting items for systematic review and meta-analyses (PRISM) to study the search strategy for recent advancements. Bioactive substances, minerals, and properties of cereal grains are impacted by various processing methods like parboiling, decoration, heating, soaking, germination, and fermentation. This paper aims to study the nutrient qualities and processing of antinutrient reduction methods, the nutritional composition of millets, their effects on consumption, and the nutritional characteristics of medicinal use. The highest dietary fiber content is in pearl millets (11.49%), followed by maize (10.20%). Millets contain carbohydrates, antioxidants, and biologically active compounds such as phenolic acids, carotenoids, flavonoids, minerals, and vitamins. The appropriate consumption of millets helps to reduce diseases like diabetes, cardiovascular diseases, inflammation, and malnutrition because of their low glycemic index, being gluten-free, and increased major nutrients. But overdose of millet consumption causes goitrogenic effects, kidney stones, thyroid dysfunction, allergic reactions, high sugar levels, and weight gain. Considering the modifications within millets’ nutritional value brought on by the process may benefit the food business, scientists, and consumers in choosing the best processing method to maximize nutrient content, boost nutrient bioavailability, and assist in promoting food and nutrition security.

## 1 Introduction

**Figure 1:**
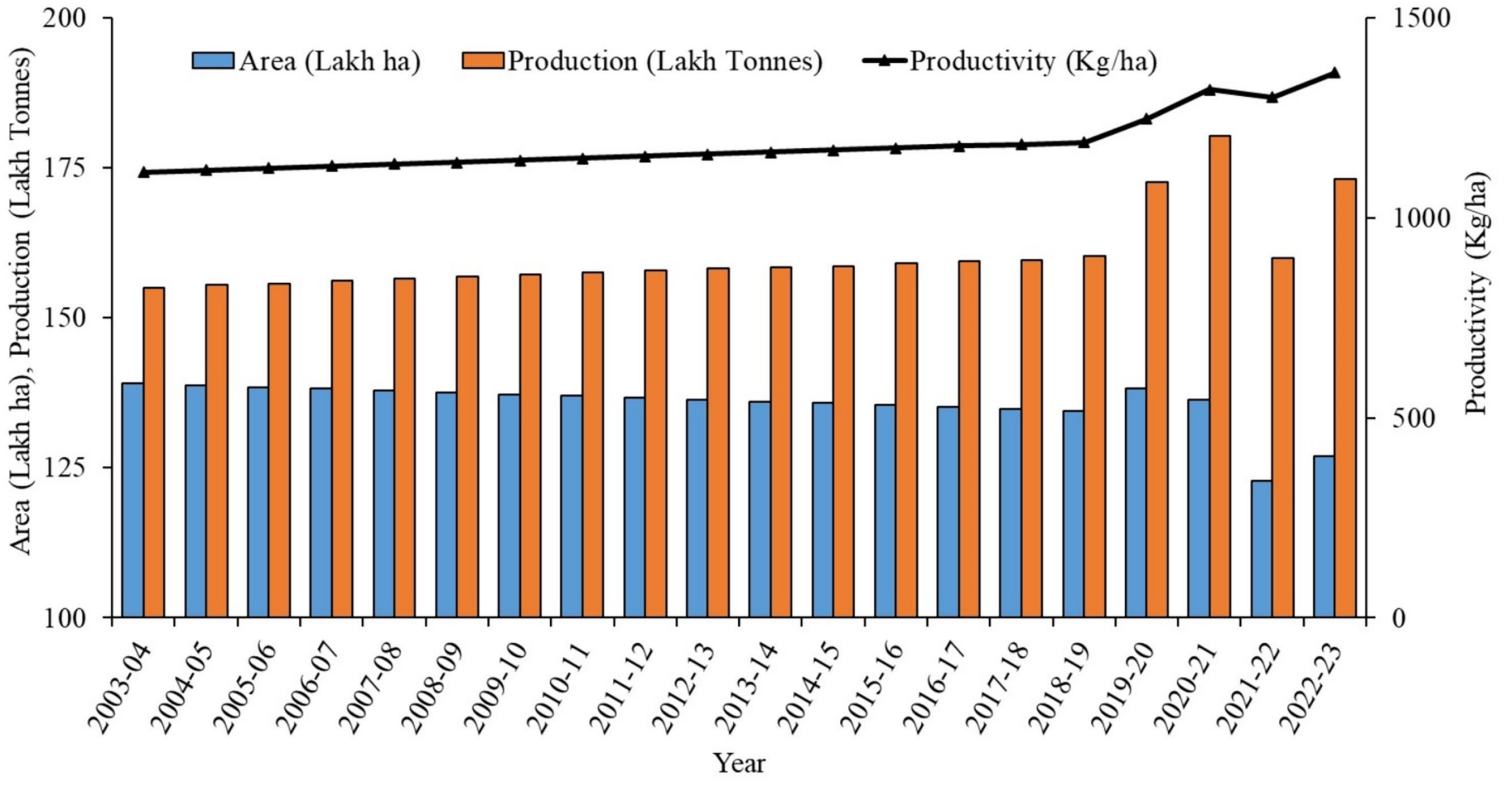
Trends in millet area, production, yield.

The United Nations declares 2023 as an “International Year of Millet,” recognizing their global importance, especially at the request of India, which leads global millet production with a 44% share, followed by China (9%) and Niger (7%) (1). Millets are primarily grown as kharif crops in rainfed regions. Belonging to the Poaceae family, it requires fewer inputs and water compared to conventional cereals. In India, between 1951 and 2022, the area under millet cultivation declined by one-third, while production and yield increased at compound annual growth rates (CAGR) of 0.2 and 1.7%, respectively, due to improved farming practices (Figure 1) (2, 3).

**Figure 2:**
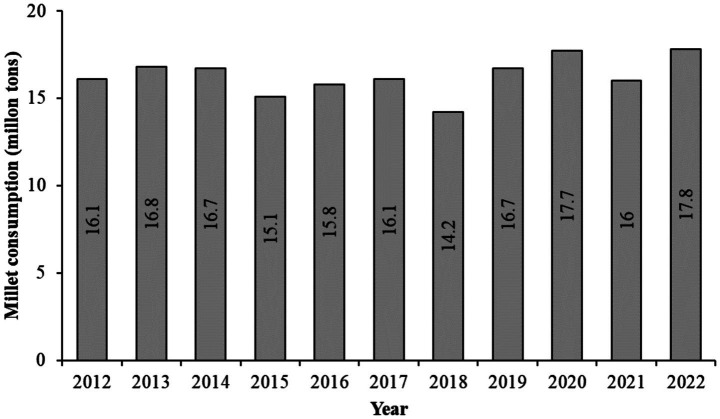
Millet consumption in India from 2012 to 2022 (4).

Millet consumption also rose significantly between 2012–13 and 2022–23 (Figure 2) (3, 4). Once regarded as coarse grains, millets are now recognized as “nutri-cereals” and are often termed “future crops” due to their resilience against pests and diseases and their adaptability to the arid and semi-arid conditions of Asia and Africa (5). Key millet species contributing to food security include sorghum (*Sorghum bicolor*), finger millet (*Eleusine coracana*), pearl millet (*Pennisetum glaucum*), teff (*Eragrostis tef*), kodo millet (*Paspalum scrobiculatum*), proso millet (*Panicum miliaceum*), little millet (*Panicum sumatrense*), foxtail millet (*Setaria italica*), and fonio (*Digitaria exilis*) (5). After decades of neglect, millets are making a strong resurgence in Indian agriculture. Despite being the world’s largest millet producer and second in rice and pulses, India ranks second globally in childhood malnutrition, with over one-third of the world’s malnourished children (6). Concurrently, rising rates of obesity and diabetes have created a dual burden of malnutrition (7). Millets, often labeled “superfoods,” are three to five times more nutrient-dense than staple cereals like rice, wheat, and maize and are naturally gluten-free (8). Enhancing millet consumption presents a sustainable strategy to strengthen national nutrition and food security while mitigating the rising prevalence of metabolic disorders.

The significant characteristics of millets include hypolipidemia, a low glycemic index, and antioxidative abilities. Millets are used to prepare noodles, soups, hard beverages, pancakes, and cereal porridges all over the world due to their nutritional benefits. Considering these into account, the various health benefits of millets are discussed in Table 1.

**Table 1:** Health benefit of various millets.

Millet	Health benefit	References
Foxtail millet	Lowers the chance of colon cancer. Reduces cholesterol and has anti-diabetic properties. Reduces the liver damage caused by ethanol.	(12, 88)
Pearl millet	The gluten-free characteristic prevents celiac disease. By decreasing Shigella pathogenicity, immunity increases.	(89, 90)
Finger millet	Minimize the risk of soft tissue injury and speed up its recovery period. Lower the risk of cardiovascular disease by lowering plasma triglycerides.	(24)
Kodo millet	Reduce the glycemic index and reduce the incidence of diabetes, in addition to having antioxidant qualities.	(91)
Proso millet	Gluten-free features may help avoid celiac disease. A low-glycemic index (GI) meal lowers type 2 diabetes risk.	(92, 93)
Little millet	Polyphenol content aids in the prevention of a number of metabolic diseases. Barnyard millet Colorectal cancer risk is reduced by destroying apoptotic cells.	(91, 94)
Barnyard millet	Diabetes is improved by inhibiting protein glycation and glycoxidation.	(13, 95)

Millets are nutritionally rich grains, offering significant amounts of vitamins and minerals. They serve as excellent sources of energy, dietary fiber, resistant starch, and slowly digestible carbohydrates, which help prolong glucose absorption and promote satiety (9, 10). Compared to major cereals, millets possess a superior fatty acid profile and are rich in essential sulfur-containing amino acids like cysteine and methionine, vital for protein synthesis (9, 11). While lysine and tryptophan levels vary among millet varieties, these grains are consistently abundant in minerals such as calcium (Ca), phosphorus (P), magnesium (Mg), manganese (Mn), potassium (K), and iron (Fe), along with vitamins E and B.

The dense nutrient profile of millets contributes to a wide range of health benefits, including reduced risk of cancer (12, 13), obesity, diabetes (14), cardiovascular disease (15), gastrointestinal disorders (16), migraines (4, 17), and asthma (5, 17). Their high fiber and low glycemic index make them particularly suitable for managing hyperglycemia in diabetic individuals (17). A valuable source of essential nutrients, millets hold potential in addressing dietary deficiencies, especially in developing and low-income regions (18). Despite these advantages, millet consumption remains limited in countries like India. However, increasing interest from startups and nutrition-focused enterprises is helping promote their use by enhancing accessibility and generating employment.

Millets require pre-consumption processing to eliminate inedible parts, enhance shelf life, and improve nutritional and sensory qualities. Primary methods like dehulling, soaking, germination, roasting, drying, and milling make millets edible (19), while secondary or modern techniques such as fermentation, parboiling, puffing, baking, and extrusion are used to produce value-added products. Although these processes improve digestibility and nutrient availability, they may also cause nutrient losses (20). This paper aims to study the nutrient qualities and processing of antinutrient reduction methods, the nutritional composition of millets, their effects on consumption, and the nutritional characteristics of medicinal use.

### 1.1 Systematic review methodology

**Figure 3:**
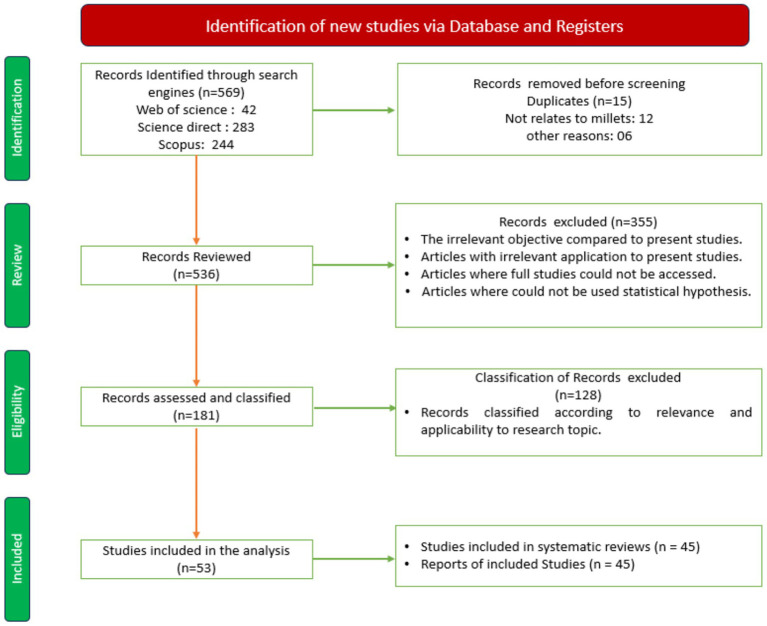
PRISMA flow chart.

PRISMA systematic review process was used to comprehensive study of selected bibliographic methodology from various electronic databases, including Web of Science, ScienceDirect, and Scopus (21). Selection performed throughout the content search and filtering process can be readily verified and justified by using the PRISMA methodology, which enables accurate and repeatable procedure for documentation. Additionally, it makes it easier to identify knowledge gaps and draw reliable inferences from the data acquired. The step-by-step process was used, followed by the PRISMA systematic review process, to identify, select, and analyze the most relevant studies on the nutritional composition of millets (Figure 3). In the first step, the identification step, a comprehensive search method was developed with the help of specific keywords and Boolean operators. In the second step, using inclusion and exclusion criteria, titles, and abstracts of 569 articles were assessed manually. In the second step, two main primary groups were identified: (1) Studies included in systematic reviews (2) Reports of included studies. The final step of PISMA is inclusion; the most relevant 53 studies were selected for the systematic analysis and divided into two (1) Studies included in systematic reviews (2) Reports of included studies. A comprehensive overview of the improvements and challenges involved in millets and nutritional composition is provided by this systematic strategy, which is backed by the PRISMA diagram and ensures transparency, repeatability, and consistency in the process.

#### 1.1.1 Identification

The search strategy was developed to identify the most relevant studies addressing the Indian millets, including their nutritional composition and antinutrient reduction methods. A comprehensive search was developed by using three scientific databases: Web of Science (42), Science Direct (283), and Scopus (244). These databases were selected because of their extensive coverage in scientific and technological domains and their capacity to index excellent, peer-reviewed research (21). The combination of specific keywords such as “nutritional composition,” “vitamin,” “mineral,” “carbohydrate,” and “protein,” etc., was selected to make the identified studies relevant. Additionally, search strategy was broadened or narrowed by using Boolean operators (AND, OR).

The search was restricted to studies published between 2015 and 2025 to encompass the most recent and relevant developments in these domains. A total of 569 research papers 42 from Web of Science, 283 from ScienceDirect, and 244 from Scopus were found in the first search, ensuring extensive coverage of the state of knowledge in this field of research.

#### 1.1.2 Study review

The present studies were selected in two stages: The first stage included titles and abstracts that were reviewed, and then the entire texts of any potentially pertinent research were thoroughly assessed.

#### 1.1.3 Inclusion and exclusion criteria

The present study’s inclusion criteria were as follows: (1) experimental studies of the various Indian millets and their nutritional properties for healthy diets; (2) different processing methods for antinutrient reduction in millets; and (3) research published in peer-reviewed scientific journals between 2015 and 2025. On the other hand, exclusion criteria were developed to eliminate studies that were not scientific (such as reviews of literature or opinion articles) or the studies did not provide specific experimental outcomes. The studies that were duplicate or lacked complete access were also excluded.

#### 1.1.4 Application criteria

The present studies were meticulously planned to identify the most relevant, high-quality research on Indian millets and their nutritional composition for a healthy diet. There are various steps taken into consideration for reduction in biases, error, and scientific accuracy.

In the first stage (identification), a comprehensive literature search was conducted, yielding a total of 569 articles related to Indian millets, their nutritional composition, and antinutrient reduction methods. The databases searched included Web of Science (42 records), ScienceDirect (283 records), and Scopus (244 records). Following the removal of duplicate records (*n* = 15), articles unrelated to millets (*n* = 12), and those excluded for other specific reasons (*n* = 6), a total of 536 articles were retained and compiled using Microsoft Excel for further screening and analysis.

Throughout the review process, inclusion and exclusion criteria were used for the titles and abstracts of the 523 papers. Every work was thoroughly reviewed to confirm its applicability to the Indian millets and their nutritional composition for a healthy diet. Consequently, 181 papers were left for the review process after 355 were eliminated due to their irrelevant objectives from the planned study, lack of empirical data, unrelated application, and failure to access the full text.

In the eligibility stage, a total of 181 scientific studies were examined and categorized based on their relevance and applicability to the study topics. The studies were divided into two categories during the final inclusion stage: studies were included in systematic reviews (45) and reports of included studies (45). The PRISMA flow diagram was illustrated to ensure reproducibility and transparency.

## 2 Nutritional composition of millets

Millets are nutrient-dense grains rich in carbohydrates, proteins, dietary fiber, essential minerals, vitamins, and bioactive phytochemicals, offering nutritional value comparable to staple cereals like rice, wheat, and maize. Average, they provide 320–370kcal per 100 grams (Table 2). Millets contain 65–75% carbohydrates and higher levels of non-starch polysaccharides and dietary fiber than traditional grains, promoting gastrointestinal health, lipid metabolism, and glycemic control. Their low gluten content and glycemic index make them ideal for individuals with celiac disease or diabetes. Additionally, millets are abundant in phytochemicals such as phytosterols, polyphenols, phytocyanins, lignins, and phytoestrogens, which possess antioxidant and immunomodulatory properties and aid in detoxification. These compounds contribute to the prevention of chronic diseases, including cancer, type 2 diabetes, and cardiovascular disorders (4). The nutritional profile of major Indian millets is outlined below and detailed in Table 2.

**Table 2:** Comparative nutritional profile of millets and major cereals (per 100 g).

Grain	Energy (Kcal)	Protin (g)	Carbohydrate (g)	Starch (g)	Fat (g)	Dietary fiber (g)	Mineral (g)	Ca (mg)	P (mg)	Mg (mg)	Zn (mg)	Fe (mg)	Thiamine (mg)	Riboflavin (mg)	Niacin (mg)	Folic acid (mg)
Sorghum	334	9.97	67.7	59	1.73	10.22	1.6	28	274	133	2.96	3.95	0.35	0.14	2.1	39.4
Pearl Milet	348	10.96	61.8	55	5.43	11.49	2.3	27	289	124	2.76	6.42	0.25	0.20	0.86	36.11
Finger millet	321	7.16	66.8	62	1.92	11.18	2.7	364	210	146	2.53	4.62	0.37	0.17	1.3	34.7
Kodo millet	332	8.92	66.2	64	2.55	6.39	2.6	15	101	122	1.65	2.34	0.29	0.20	1.49	39.99
Little millet	346	10.13	65.6	56	3.89	7.72	1.7	16	130	91	1.82	1.26	0.26	0.05	1.29	36.2
Proso millet	341	12.50	70.4	–	1.10	2.20	1.9	14	206	153	1.40	0.80	0.41	0.28	4.5	–
Foxtail millet	331	12.30	60.1	–	4.30	8.00	3.3	31	188	81	2.40	2.80	0.59	0.11	3.2	15
Barnyard millet	307	6.20	65.6	–	2.20	9.80	4.7	20	280	82	3.00	5.00	0.33	0.10	4.2	–
Maize	334	11.5	64.7	59	3.6	10.2	1.5	8.9	348	–	–	2.49	–	–	–	–
Wheat	322	10.59	64.7	56	1.47	11.23	1.5	39	315	125	2.85	3.97	0.46	0.15	2.68	30.1
Rice	356	7.94	78.2	71	0.52	2.81	0.6	7	96	19	1.21	0.65	0.05	0.06	1.69	9.32

Source: NIN (96) and IIMR (97).

### 2.1 Proso millet

It offers a superior nutritional profile compared to other staple grains, with higher levels of minerals and dietary fiber (Table 2). It is rich in essential vitamins and minerals such as Fe, Ca, K, P, Zn, Mg, B-complex vitamins, niacin, and folic acid. Proso millet also contains greater amounts of essential amino acids, except lysine, which remains the limiting amino acid. The essential amino acid index is approximately 49% higher than wheat (22). Additionally, studies have shown that proso millet-based products produce a significantly lower glycemic response than wheat and rice, highlighting their potential role in glycemic management.

### 2.2 Pearl millet

It offers energy levels comparable to common cereals but lower carbohydrate content. Its high amylose starch (20–22%) and substantial insoluble fiber contribute to a lower glycemic response. Being gluten-free and rich in prolamins, it is suitable for individuals with gluten intolerance. Although it has a generally favorable amino acid profile, it is limited in lysine, threonine, tryptophan, and sulfur-containing amino acids (23). Pearl millet also provides essential fatty acids such as alpha-linolenic acid, eicosatetraenoic acid (EPA), and docosahexaenoic acid (DHA), along with important micronutrients like Fe, Zn, Cu, K, Mg, P, and Mn (23).

### 2.3 Kodo millet

It offers an energy content comparable to other millets and grains. Although its protein content is relatively lower than most millets except finger millet, it still serves as a valuable gluten-free protein source (Table 2). It is particularly rich in B-complex vitamins such as B6, niacin, and folic acid, and essential minerals like iron, calcium, magnesium, potassium, and zinc. Due to its high digestibility, kodo millet is well-suited for formulating food products for infants and the elderly (24).

### 2.4 Foxtail millet

It is relatively more expensive than other cereals, foxtail millet offers superior nutritional benefits compared to common staples like wheat and rice. It is rich in dietary fiber, resistant starch, essential vitamins, minerals, and key amino acids, excluding lysine and methionine. Notably, foxtail millet has the highest protein content among all millets (Table 2) and contains significant amounts of stearic and linoleic acids, which support healthier lipid profiles (8, 12).

Finger millet: Finger millet has the highest carbohydrate content among millets, but unlike common cereals like rice and wheat, its carbohydrates have a lower glycemic index due to the presence of slowly digestible starch, dietary fiber, and resistant starch (25). Although its protein content is relatively lower than that of other millets (Table 2), it offers a favorable amino acid profile, particularly rich in threonine, lysine, and valine. Also, it is a good source of micronutrients such as Ca, Fe, Mg, K, and Zn, along with B-complex vitamins including niacin, vitamin B6, and folic acid.

### 2.5 Little millet

Little millet has a nutritional profile comparable to other cereals and millets, containing around 8.7% protein with a well-balanced amino acid composition. It is particularly rich in lysine and sulfur-containing amino acids like cysteine and methionine, which are generally low in common cereals (26). Its high levels of dietary fiber, resistant starch, and slowly digestible starch contribute to a low glycemic response (27). Additionally, little millet provides essential micronutrients such as niacin, Fe, and P. Recently, a range of value-added products has been developed to harness its nutritional and health benefits.

## 3 Antinutrient reduction methods

Antinutrients are phytochemicals that plants spontaneously make as a form of protection. The antinutritional effects lead to decreased bioavailability of nutrients and their utilization (28). The presence of antinutritional factors and certain chemical compounds in raw plant-based foods can pose health risks, including bloating, impaired nutrient absorption, and micronutrient deficiencies, potentially leading to malnutrition. Millets, despite their high nutritional value, contain elevated levels of antinutrients compared to staple cereals like wheat and rice. Common antinutrients in plant-based foods include tannins, phytates, oxalates, and enzyme inhibitors such as trypsin and chymotrypsin inhibitors (29). Finger millet, for example, has significant amounts of polyphenols, tannins (0.61%), phytates (0.48%), oxalates, and trypsin inhibitors, which can impair micronutrient absorption and protein digestion (4). However, certain antinutritional compounds, particularly polyphenols, have recently been recognized for their nutraceutical potential due to their antioxidant properties. While some secondary metabolites function as antinutrients, others exhibit pharmacological activity and are being explored for therapeutic use. To reduce antinutrient effects and improve nutrient bioavailability, processing methods such as decortication, soaking, heating, germination, and fermentation are commonly used. These treatments enhance the absorption of essential minerals like Ca, Fe, and Zn, and also improve protein digestibility, thereby increasing the overall nutritional value of millet-based foods (Table 3) (19).

**Table 3:** Studies carried out by various researchers on antinutrient reduction method and their effect on processing method.

Processing method	Millets	Experimental condition	Experimental outcomes	Effect of processing method	Disadvantages	References
Parboiling	Finger millet	70°C at 10 to 24 h	Reduces phytic acid content by 20 to 30%. Facilitates efficient separation of the endosperm from the bran. Increase in residual starch content.	Maximum grain recovery with lower nutrient loss. Reduces phytic acid levels and facilitates the separation of the endosperm from the bran. Decreases the glycemic index while enhancing residual starch content.	Altered texture and cooking quality. Sensory change. Energy intensive. Nutrient specificity and loss.	(31, 32)
Dehulling/ Decortication	Pearl	30 ± 2°C for 14 h	Protein content increased. Decrease phytic acid content from 943 to 380 mg/100 g (59.5% reduction).	Removal of husk from millets. Millets are usually dehulled or decorated by using a mortar and pestle or mill stone, resulting in fatigue during processing operations.	Significant Nutrient loss. Incomplete antinutrient removal. Variable efficacy. Grain breakage.	(33)
Heating	Proso	Pan and microwave cooking	Elevated carbohydrate content but reduced fat content. Protein content in pan and microwave cooking was increased and decreased, resp.	Change of fat content, smell, texture, color, nutrient composition, flavor depends on heating method.	Loss of heat sensitive nutrients. Thermal stability of certain anti-nutrients. Reduced protein digestibility and mineral. Mineral losses. Negative effects on organoleptic properties. Time and energy consumption.	(101)
	Little	Pan and microwave cooking.	Elevated carbohydrate content but reduced fat content. Protein content in pan and microwave cooking was increased and decreased, resp.			(101)
	Pearl	Roasting 150 °C for 5 min.	Enhanced the proportion of all polyphenols that are bio-accessible for native and roasted sample by 73.2 to 78.1%. Elevated bio-accessible flavonoid.			(102)
		Microwave heating	Decreased bio-accessible of phenolic content.			
	Kodo	Boiling 95 to 100 °C for 25 min.	Elevated water absorption capacity and porosity. Decreased starch yield.			(103)
Soaking	Pearl	Soaking for 24 h	Protein content elevated due to release of stored nitrogen grains. With sprouting fat and crude fiber increases. Sprouting reduces Co, Cr, Cu, Zn, Fe, Na, K.	Increases the grains’ capacity to absorb moisture and enhances the bioavailability of zinc quickly and uniformly. Inhibition of trypsin action. At 25 °C, over a 12-h period, reductions were observed in several antinutritional factors: trypsin inhibitor activity (TIA), oxalates, polyphenols, and saponins decreased by 33%, tannins by 20%, and phytate levels declined from 39.47 to 24.17%.	Loss of water-soluble nutrients. Limited and variable effectiveness. Time consuming process. Risk of souring. Lack of control on process parameters.	(30, 104–106)
Germination	Foxtail	Germinated for 46.5 h	Protein content increased (13.37 g/100 g) compared to raw (10.60 g/ 100 g). Increase minerals like Fe, Mg, Ca, Na. The fat content is reduced from 3.86 to 2.78 g per 100 grams.	Reduces antinutritional content and increases the mineral biodiversity. Substantially decreased amylase activity, trypsin inhibitor, phytates by 54% and tannin by 65%.	Time intensive process. Risk of spoilage and mold growth. Loss of dry matter and nutrients. Variable effectiveness across antinutrients. Potential for antinutrients resynthesis. Decline of bio accessibility of some compounds. Potential loss of water-soluble nutrients.	(30, 54, 55)
		Germination at room temp.	Protein, dietary fiber and phenolic content increased by 29.72, 58.02 and 77.42%.			^74,^
	Kodo	38.75 °C for 36 h	The mineral content, protein, and dietary fiber increase, while total carbohydrate levels decline.			(107)
	Pearl millet	Sprouting at 72 h for room temperature.	Decrease fat and Ash content. After germination iron and calcium content increases.			(108)
Fermentation	Pearl var	*Lactobacillus Plantarum*	After 96 h of fermentation, the protein content increased from 8.7 to 20.54% in the sample with a starter culture and to 20.21% in the naturally fermented sample. Reduces carbohydrate with increase of soluble sugar. The lipid content decreased significantly from 10.34 to 0.34% in the starter culture sample and to 0.74% in the naturally fermented sample.	• Enhances the biological value (BV), net protein utilization (NPU), and some vitamins. • Enhances dietary value and flavor. • A decrease of 20, 52, and 32%, respectively, in phytates, tannins, and trypsin inhibitors.	Time consuming. Risk of contamination. Variable quality and consistency. Sensory changes. Incomplete removal of all nutrients. By product accumulation.	(109, 110)
	Foxtail	Fermentation followed heat moisture treatment.	Crude protein content improves. Total carbohydrate level decrease. Increases nutritional quality of starch.			(54)
		Fermentation using *L. paracasei* Fen032 strain.	The crude protein content increased by 20.51% in the fermented sample. The total carbohydrase content decreased by 74.02%.			(111)

### 3.1 Parboiling

Parboiling is a hydrothermal pre-treatment applied to millets prior to decortication to improve grain recovery and reduce kernel breakage. The process comprises three sequential steps: soaking, steaming, and drying (30). Soaking is performed at approximately 70 °C for 10–24 h to achieve adequate moisture uptake, during which prolonged soaking can reduce phytic acid content by 20–30% (31). The soaked grains are then steamed in autoclaves or steamers to gelatinize endosperm starch, followed by tray or open sun drying to reduce the moisture content to about 10%. Parboiling facilitates separation of the endosperm from the bran and promotes nutrient migration from the seed coat into the kernel. Varadgaraju and Ganesan (32) reported that parboiling reduces the glycemic index and increases resistant starch content in millets, thereby enhancing their potential as prebiotic ingredients.

### 3.2 Decortication/dehulling

It is the process of removing the outer part (pericarp) of the grains. Husk in pearl millet is 1.5 to 29.3%. Conventionally, decortication was carried out by hand using a mortar and pestle; however, a rice huller rice milling machine was used. El Hag Mardia (33), observed that the decortication decreased the total polyphenolic content (TPC) and phytic acid in pearl millet by 9 and 53%, respectively. On the other hand, Pal et al. (34) reported that the phytic acid concentration of lentils decreased by 52.63 to 56.00%. In a recent experiment conducted by Himanshu et al. (35), this method reduced the levels of little millet, barnyard millet, kodo millet, and common millet by 39, 23, 25, and 12%, respectively.

### 3.3 Heating

Heating methods such as roasting, boiling, cooking, and autoclaving significantly reduce antinutritional factors in millets and legumes. Roasting was shown to lower tannins, phytates, trypsin inhibitors, and protease inhibitors by 74.6, 28.4, 98.3, and 97.5%, respectively, while cooking reduced them by 42, 75.8, 95.8, and 95.8% (36). Sade (37) reported that roasting decreased tannins from 0.51 to 0.29 mg/100 g and phytates from 0.21 to 0.11 mg/100 g. Additionally, cooking led to an increase in total phenolic content (TPC) (38). Abdelrahman and ElMaki (39) observed reductions of 6–10% in phytic acid and 5–8% in polyphenols following heat treatment. In germinated horse gram, trypsin inhibitor activity was reduced by 26.79%, while in cooked lentils, it declined by 80.51–85.41% (34, 40).

### 3.4 Soaking

It is a simple and widely used pretreatment before germination, cooking, or further processing, primarily to reduce antinutritional factors (41). Typically conducted for 12 to 18 h, soaking effectively decreases levels of soluble phytic acid and protease inhibitors. Roy et al. (42) studied five chickpea varieties (Virat, Annigeri, IC68966, BGM 408, and CUML4) and observed reductions in tannins and phytic acid levels ranging from 16.90–23.28% to 15.19–17.78%, respectively. Fernandes et al. (43) noted that discarding soak water helps remove significant amounts of antinutrients, mainly by leaching out polyphenols. Singh et al. (44) further reported that combined treatments like soaking, germination, microwave processing, and fermentation reduced polyphenol content by up to 70%.

### 3.5 Germination

Germination, an active metabolic phase, reduces antinutritional factors while altering the chemical composition of grains and legumes. This process enhances their nutritional value by decreasing compounds that hinder nutrient absorption (45). Sokrab et al. (46) reported that germination decreases phytic acid content while increasing polyphenol content. Germination increased TPC by 5.57% in peanuts and lowered it by 25.96% in soybeans (47). In different pearl millet cultivars, the phytic acid content varies from 588 to 1382 mg/100 g. Pearl millet germinated at 24 h at 30 °C; the phytate above 50% occurs. Handa et al. (48) reported that the germination lowers tannin content and TPC from 199.85 to 100.30 mg/100 g and 134.71 to 65.19 mg GAE/100 g. A reduction of 60% was found after germination (49).

### 3.6 Fermentation

Fermentation helps to increase the nutritional values and antioxidant capabilities of legumes. It improves digestion and absorption of protein while removing some natural antinutritional elements, such as phytic acid. Fermentation plays a key role in food processing by increasing the flavor, texture, and aroma and helps improve the overall palatability and nutritional appeal of food (50). It enables the preservation of large quantities of food through various microbial pathways, including lactic, acetic, ethanolic, and alkaline fermentation. This process also enriches foods with essential vitamins, proteins, and amino acids, thereby improving their biological quality. Additionally, fermentation contributes to the reduction of natural toxins and decreases the fuel and time required for cooking, making it an energy-efficient and health-promoting processing method.

Rasane et al. (51) reported that the non-fermented, roasted, and germinated pearl millets have a higher phytic acid content than the fermented, roasted, and germinated samples. The amount of phytic acid in pearl millet decreased after being fermented for 24 hours (52). A mixture of *S. cerevisiae*, *S. diastaticus*, *L. brevis*, and L. fermentum was used to ferment germinated pearl millet buds at 30 °C for 72 h, which resulted in 88.3% phytate concentration (53).

## 4 Effect on the nutritional properties of millet processing

### 4.1 Protein

Millets are high in protein, and in comparison, to animal proteins, these are excellent plant proteins with small quantities of saturated fat. Antinutrients hinder protein digestion; thus, lowering antinutrient levels is essential. Simple procedures, including dehulling, grinding, soaking, and heating, rneduce antinutrient levels while increasing protein digestibility *in vitro*. The effect of different processing procedures on the protein digestibility of foxtail millets has been investigated (54). The protein quality of foxtail millet improves with processing methods such as alkaline boiling, fermentation, germination (40 h at 25 °C), and popping. In proso millet, pan-frying increased protein content by 9.5% (20). Protein digestibility in cereals, millets, and legumes is notably enhanced during germination and fermentation, largely due to the synthesis of additional amino acids (55). In foxtail millet, germination boosts protein levels, while similar increases were observed in two pearl millet cultivars Gadarif (11.4 to 13.2%) and Gazeera (14.4 to 16.3%) (56). Germination has also been shown to raise total protein content in pearl millet from 14 to 26%, and extended sprouting (96 h) led to increased protein in proso millet (57, 58).

### 4.2 Carbohydrates

Carbohydrates make up 60–75% of millets, with foxtail millet containing the least and little millet the most (Table 3). Like other cereals, millets are rich in starch; however, the bioavailability of their carbohydrates can vary depending on processing methods such as soaking, sprouting, pressure cooking, and autoclaving (4). During germination, an increase in carbohydrate content has been observed in foxtail millet, likely due to the reduction in crude protein, fat, ash, and moisture, which shifts the overall composition toward carbohydrates. Specifically, foxtail millet showed a 1.29% increase in carbohydrate content following germination (59). In contrast, pearl millet flour demonstrated a slight increase in carbohydrate content within the first 24 to 48 h of germination, followed by a slight decline at 72 h (60). Studies on the effects of fermentation and germination in pearl millet revealed that germination increased total soluble sugars while decreasing non-reducing sugars. Homogenization and autoclaving of germinated slurry significantly increased soluble sugar levels while reducing starch content (61).

### 4.3 Dietary fiber

Millet bran is a valuable source of dietary fiber, primarily composed of indigestible polysaccharides. However, decortication or dehulling significantly reduces fiber content by removing the bran layer. Studies suggest that dehulling between 12and 30% effectively eliminates the outer kernel while minimizing fiber loss, whereas removal beyond 30% leads to a substantial decline in fiber levels (62). Sharma and Niranjan (54) reported that milling reduced insoluble fiber components such as lignin, cellulose, and hemicellulose in foxtail millet compared to whole flour. While germination increased fiber content, treatments like fermentation and acid pre-extrusion reduced it further to 0.9–1.4 g/100 g. Additionally, high-temperature extrusion exacerbates fiber degradation. As dietary fiber from the bran plays a crucial role in preventing conditions like type 2 diabetes and constipation, excessive polishing should be avoided. Promoting whole millets and their byproducts is essential to preserving their nutritional value in diets.

### 4.4 Minerals

Millets are abundant in essential minerals, such as K, Mg, Fe, Ca, and Zn, along with various vitamins primarily located in the aleurone layer, germ, and pericarp (4). Soaking millet grains before cooking effectively reduces antinutritional compounds and enhances mineral bioavailability. However, soaking may lead to a decline in Fe and Zn levels due to leaching into the soaking medium (63). Despite this, soaking improves *in vitro* mineral solubility by about 2–23%. Optimal conditions, such as soaking in hot water (45–65 °C) at pH 5–6, further increase mineral availability and significantly reduce phytic acid content. 88 Germination and fermentation also modify the mineral composition of millet flours (64). Specifically, germination in foxtail millet enhances nutrient accessibility by breaking down antinutrients like saponins and polyphenols that otherwise hinder mineral absorption (54, 55, 64).

### 4.5 Vitamins

Polishing or debranning millets reduces their nutritional quality by removing the bran and germ, which are rich in essential vitamins. Nonetheless, millets retain a nutritional advantage over wheat, sorghum, and maize, particularly in their higher content of vitamins (thiamine, riboflavin, niacin, and folic acid), lipids, proteins, and minerals, primarily concentrated in the aleurone layer, germ, and pericarp (Table 3) (65). Germination and fermentation significantly alter the vitamin composition of millets. For instance, fermentation has been shown to enhance thiamine content in pearl millet. Conversely, certain processes lead to nutrient losses; i.e., decortication of little millet reduces vitamin E content by approximately 67% (66). Milling, which removes the bran layer, results in substantial losses of vitamins. In pearl millet, milling leads to a marked decline in B vitamins and a modest reduction in vitamin E. Similarly, milling and sieving of finger millet flour result in decreased levels of thiamine (from 0.552 to 0.342 mg/100 g) and riboflavin (from 0.243 to 0.196 mg/100 g) (67). However, germination has been found to increase vitamin C levels in finger millet, rising from 0.04 to 0.06 mg/100 g (68). Overall, milling and dehulling processes tend to deplete vitamin content due to the concentration of these nutrients in the grain’s outer layers. Enhancing vitamin availability can be achieved through germination and by utilizing germinated millet-based products.

### 4.6 Fats

Fats are essential for energy provision, brain development, and the absorption of fat-soluble vitamins (A, D, E, and K). In millets, fat content is significantly influenced by processing methods. Germination reduces fat levels, as observed in foxtail millet flour, where values declined to 4.4 and 3.6% due to the utilization of stored fats for energy. High-pressure soaking also led to a 27.98% reduction, attributed to enzymatic activity releasing soluble nutrients (69). Similarly, malting pearl millet for 24 h reduced fat content from 6.34 to 5.55% (70). These reductions are mainly due to enzymatic breakdown and energy metabolism during processing. Additionally, treatments like cooking, popping, and milling contribute to fat loss. While methods like soaking, germination, and malting are effective for developing low-fat millet products, high-temperature processes may negatively impact fat quality and sensory characteristics.

## 5 Comparative study of cost effectiveness and efficacy of antinutrient reduction methods

The concentration effects must also be considered, since the majority of metabolism is safe when consumed in small amounts. Antinutrients are used as active ingredients in food and beverages. When consumed in small quantities, these substances, including phytic acids, phenolic acids, and saponins, have been found to reduce cholesterol and glucose levels (29). Additionally, saponin is reported to improve liver function and prevent osteoporosis. Moreover, phenolic substances with anti-cancer effects include phytic acids, saponins, and protease inhibitors (71). On the other hand, tannins have been found to have antiviral, antiparasitic, antibacterial, antioxidant, anticancer, immunoregulatory, and cardiovascular protective properties. It is essential to be aware of the ingredients in food items and to exercise when consuming them. Antinutrients have several advantageous effects and are useful in the treatments of many diseases (71, 72). The consumption of a proper quantity of millets has positive effects even when it is not always nutritious.

The various methods used for antinutrients reduction was illustrated in Table 4. From the table it is observed that parboiling and soaking are the most economical methods to reduce antinutrients. Also, the heating, fermentation, germination, parboiling are found to be highly efficient methods. Overall, combination processing methods produce better results than the single methods.

**Table 4:** Comparative study of cost effectiveness and efficacy of antinutrient reduction methods (98–100).

Methods	Major antinutrients reduction	Cost effectiveness	Efficiency
Parboiling	Trypsin, lectins, oxalates.	High	Moderate to high
Dehulling/Decortication	Phytic acid, tannins	Moderate	High
Heating	Trypsin, tannins	Moderate to high	Very high
Soaking	Phytic acid, tannins, water soluble ANFs	Very high	Moderate
Germination	Phytic acid, Polyphenol, enzyme inhibitors.	Moderate	High
Fermentation	Phytic acid, tannins, saponin, enzyme inhibitors, α-galactosides.	Moderate	Very High

## 6 Nutritional characteristics of various millets and medicinal uses

Currently, awareness of millets as a nutrient-dense and healthy dietary source has grown significantly. Millets provided significantly higher nutritional values as compared to commonly grown and consumed food grains like wheat, rice, and maize. The various Indian millets, micronutrients, vitamins, minerals, (1) and their uses (Table 5): Generally, millets contain vitamins, antioxidants, biologically active compounds, magnesium, zinc, calcium, iron, phosphorous, and several other minerals. These millets, commonly called “nutricereals” because of their nutrients, reach ancient foods (73–75). There are several functional uses of millets, like sustaining the various bodily functions, including immune system function and energy metabolism (76–78). Generally, carbohydrate content in millets ranges from 50 to 88%, of which 60–75% is starch, followed by non-polysaccharides (15–20%) and free sugars (1–3%), depending upon the varieties, topography, crop management, and agroclimatic conditions, as compared to wheat (68–75%), rice (75.9–82.7%), and maize (63.19–74.5%), respectively (79).

**Table 5:** Nutritional characteristics of various millets and medicinal uses.

Sr. No.	Millets	Common name	Micronutrient/Phytochemical	Vitamins	Mineral	Uses	Bad effect	Ref
1	Sorghum	Jawar	B-carotene, folic acid, riboflavin, fiber thiamine, tannins, flavonoids, phenolic acids	Vitamin B1, B2, B9	Calcium, potassium, phosphorous, iron, sodium and zinc	Lowers obesity, anti-carcinogenic, heart diseases, arthritis, esophageal cancer, hunger	Digestive issues, reduced mineral absorption, allergic reaction, thyroid function.	(112, 113)
2	Pearl millet	Bajra	Unsaturated fatty acids	Vitamin B, Complex and folate	Calcium, Copper, magnesium, iron, and zinc	Breathing problems, headaches, Anemia	Goitrogenic effects, Kidney stone, Thyroid dysfunction, allergic reaction.	(114)
3.	Finger millet	Ragi	Dietary Fibers and Protein	Vitamin A and Vitamin B	Calcium and Phosphorus	Diarrhea, gastrointestinal, cholesterol level in blood, etc	Kidney stone, Thyroid function, mineral absorption, digestive discomfort.	(115, 116)
4	Kodo millet	Kodra	Highest amount of dietary fiber, substantial protein content, lower fat and lecithin	Folic acid, niacin, pyridoxine	Calcium, magnesium, potassium, iron, and zinc	Enhancing neurological system, postmenopausal women with dyslipidemia, diabetes, cardiovascular condition	Digestive issues, thyroid issue, mineral absorption. Constipation.	(117)
5	Little millet	Gajrao	Dietary Fiber (37–38%), protein, apeginin	Vitamin B	Phosphorus, iron	Heart diseases, diabetes, gluten allergy, increased cholesterol and anticancer properties.	Digestive delayed, thyroid issue, blood sugar, allergic reaction, reduce essential nutrients.	(118, 119)
6	Proso millet	China	Caffeic acid, chlorogenic acid, ferulic acid, syringic acid.	Vitamin B	Calcium, phosphorous, magnesium, sodium, iron, and zinc	Diabetes, Heart diseases	Thyroid interference, digestive discomfort, reduced mineral absorption, blood sugar spikes, allergic reaction.	(23)
7	Foxtail millet	Kangni	Protein, dietary fiber, catechin, quercetin, apigenin.	Vitamin B, Niacin (B_3_), Riboflavin (B_2_), Thiamin (B_1_)	Calcium, iron, Copper	Heart diseases, diabetes, dyslipidemia, antibiotic, ulcers.	digestive discomfort, thyroid function, allergic reaction, kidney stone risk.	(120–122)
8	Barnyard millet	Swank or Shyama	Carbohydrate, protein, dietary fiber, Linolic acid, palmitic acid, oleic acid, phenolic compound, flavonoids, serotonin, tricin,	Vitamin B, Niacin, B-complex vitamin, folic acid.	Calcium, iron, magnesium	Diabetes, antioxidant, anti-rheumatic, antibiotic, celiac diseases.	Delayed digestion, thyroid function, stomach upset,	(123–126)
9	Maize	Corn or Makka	Carbohydrate, vitamin, fat, fiber, Phenolic acids, flavonoids,	Vitamin B, Niacin (B_3_), Riboflavin (B_2_), Thiamin (B_1_), pyridoxine (B_6_), folic acid, Vitamin A	Calcium, phosphorous, potash, iron, and zinc	Diabetes, chronic diseases, Bone health.	Digestive problem, Blood sugar spikes, Weight gain, obesity, reduced mineral absorption, mycotoxin contamination, pellagra risk, metabolic syndrome.	(127, 128)
10	Wheat	Gehun	Phenolic acids, carotenoids, tocopherol, alkylresorcinols, benzoxaziboids, lignans, flavonoids, phenols, saponins etc.	Niacin (B_3_), Riboflavin (B_2_), Thiamin (B_1_), Folate	Calcium, phosphorous, magnesium, potassium. Manganese, iron, and zinc	Diabetes, cardiovascular diseases, antioxidant properties, malnutrition.	Celiac diseases, inflammatory bowl issues, sluggish digestion, weight gain, blood sugar spikes, skin problem, fatigue and kidney stone.	(129)
11	Rice	Chawal, Tandul, Bhat	Phenolic compound, phenolic acids, flavonoids, Tannins etc. phytates, phytosterol, carotenoids.	Niacin (B_3_), Riboflavin (B_2_), Thiamin (B_1_), Vitamin E	Calcium, phosphorous, magnesium, potassium. Manganese, iron, and zinc	Diabetes, cardiovascular diseases, weight management, digestive health cancer prevention, gluten free alternative etc.	Blood sugar spikes and diabetes issues, weight management issues, nutrient deficiency, digestive problem.	(130)

Additionally, millets contain dietary fiber, which is made of cellulose, hemicellulose, *β*-glucan, lignin, and arabinoxylans. There are several minerals contained in millets, like magnesium, calcium, potassium, phosphorous, manganese, iron, and B vitamins like folate, pantothenic acid, thiamin, riboflavin, niacin, and vitamin B_6_. Vitamins and minerals are the most important and beneficial components in the human body, as they support muscle and nerve function, hormone regulation, and the maintenance of the body’s water balance; skeletal tissues are also essential in our diets (80, 81).

Dietary fibers are complex carbohydrates with several health benefits that are difficult for the tiny intestine to break down and absorb (82). Dietary fiber has several health benefits, including improving gut health, reducing the risk of heart disease, avoiding constipation, and lowering the food glycemic index (GI) (83). The millets contain a protein level of 5–17%, as compared to wheat (12–13.9%), rice (6.8–9.5%), and maize (8–9%). The highest protein level is found in proso millet (12.50%), followed by foxtail millet (12.30%) and maize (11.50%) (Table 5). But major factors considered to be good for food consumption is dietary fiber. The highest dietary fiber found is pearl millets (11.49%) followed by maize (10.20%).

However, the appropriate dose reduces various diseases such as breast cancer, cardiovascular diseases, and inflammation (84). There are various fundamental techniques to reduce the antinutritional properties, like dehulling, soaking, germination, roasting, drying, polishing, and milling (size reduction) (85). Additionally, secondary processing methods like fermentation, parboiling, frying, malting, puffing, extrusion, baking, popping, and flaking are used to produce value-added processed food items based on millets (Table 5) (86, 87). Overall, millets increase the nutrient digestibility and consumption.

## 7 Future research direction

The consumption of millets has been studied, but there are still significant research gaps and areas for more detailed analysis. Addressing these gaps helps guide future study and provide more comprehensive views of possible effects of millets. Further research is required to elucidate the health impacts of millet consumption. Detailed investigation of specific bioactive compounds, including their bioavailability, metabolism, and interactions with physiological pathways, is essential to clarify the mechanisms underlying disease risk reduction. Additional studies are needed to determine optimal millet intake, including appropriate quantities and consumption frequencies for disease prevention and management. Comparative analyses of different millet cultivars and their nutrient profiles are also warranted. Moreover, well-designed clinical studies, particularly randomized controlled trials involving populations with conditions such as diabetes, obesity, cardiovascular disease, and gastrointestinal disorders, are necessary to validate the associations between millet consumption and health outcomes. Comparative studies evaluating processing and cooking methods are also needed to assess their effects on nutrient retention, bioactive compound preservation, and overall nutritional quality.

## 8 Conclusion

Millets are nutrient-dense grains with high levels of fiber, vitamins, minerals, and phytochemicals, offering considerable health benefits and potential in managing chronic diseases. Processing methods such as soaking, germination, and fermentation enhance their nutritional value by improving protein digestibility and mineral bioavailability, making millets effective in addressing protein-energy malnutrition, particularly in low-resource settings. Conversely, mechanical processing techniques such as decortication, milling, and dehulling can increase total protein and fiber content but may also lead to substantial losses of micronutrients and dietary fiber if not carefully controlled, as these processes often remove the nutrient-rich bran and germ layers. Therefore, careful optimization of processing techniques is essential to preserve the health-promoting properties of millets while enhancing their sensory appeal. The parboiling and soaking are the most economical methods to reduce antinutrients while heating, fermentation, germination, parboiling are found to be highly efficient methods. The appropriate and right amount of millets consumption was good for healthy diet. But overdose of millets consumption causes digestive issues, kidney stone, allergic reaction, weight gain, sugar level issue etc. Hence, appropriate amount of millets consumption was good for health and healthy diet.
